# Comprehensive profiling of transcriptional networks specific for lactogenic differentiation of HC11 mammary epithelial stem-like cells

**DOI:** 10.1038/s41598-018-30122-4

**Published:** 2018-08-06

**Authors:** Trinadha Rao Sornapudi, Rakhee Nayak, Prashanth Kumar Guthikonda, Anil Kumar Pasupulati, Srinivas Kethavath, Vanita Uppada, Sukalpa Mondal, Sailu Yellaboina, Sreenivasulu Kurukuti

**Affiliations:** 1 Department of Animal Biology, School of Life Sciences, University of Hyderabad, Hyderabad, 500046 India; 2 CR Rao Advanced Institute of Mathematics, Statistics and Computer Sciences, University of Hyderabad campus, Gachibowli, Hyderabad, 500046 India; 3 Department of Biochemistry, School of Life Sciences, University of Hyderabad, Hyderabad, 500046 India; 4 Nucleome Informatics Private Limited, 2nd Floor, Genome Block, Plot No 135, Mythrinagar Phase I, Madinaguda, Hyderabad, 500049 India

## Abstract

The development of mammary gland as a lactogenic tissue is a highly coordinated multistep process. The epithelial cells of lactiferous tubules undergo profound changes during the developmental window of puberty, pregnancy, and lactation. Several hormones including estrogen, progesterone, glucocorticoids and prolactin act in concert, and orchestrate the development of mammary gland. Understanding the gene regulatory networks that coordinate proliferation and differentiation of HC11 Mammary Epithelial stem-like Cells (MEC) under the influence of lactogenic hormones is critical for elucidating the mechanism of lactogenesis in detail. In this study, we analyzed transcriptome profiles of undifferentiated MEC (normal) and compared them with Murine Embryonic Stem Cells (ESC) using next-generation mRNA sequencing. Further, we analyzed the transcriptome output during lactogenic differentiation of MEC following treatment with glucocorticoids (primed state) and both glucocorticoids and prolactin together (prolactin state). We established stage-specific gene regulatory networks in ESC and MEC (normal, priming and prolactin states). We validated the top up-and downregulated genes in each stage of differentiation of MEC by RT-PCR and found that they are comparable with that of RNA-seq data. HC11 MEC display decreased expression of *Pou5f1 and Sox2*, which is crucial for the differentiation of MEC, which otherwise ensure pluripotency to ESC. *Cited4* is induced during priming and is involved in milk secretion. MEC upon exposure to both glucocorticoids and prolactin undergo terminal differentiation, which is associated with the expression of several genes, including *Xbp1* and *Cbp that* are required for cell growth and differentiation. Our study also identified differential expression of transcription factors and epigenetic regulators in each stage of lactogenic differentiation. We also analyzed the transcriptome data for the pathways that are selectively activated during lactogenic differentiation. Further, we found that selective expression of chromatin modulators (*Dnmt3l*, *Chd9*) in response to glucocorticoids suggests a highly coordinated stage-specific lactogenic differentiation of MEC.

## Introduction

The events of cellular differentiation, which lead the transition of a primary cell into lineage-restricted phenotype, are accompanied by activation of specific gene regulatory networks instead of activation of a single or a few genes^1,2^. The cell fate transitions experience a global transcriptional activation and repression and are regulated by spatiotemporal expression of both transcription factors^3–5^ (TFs) and epigenetic regulators^6^ (ERs). The transition of mammary epithelial cells (MEC) to lactogenic cells is considered as a model system to comprehensively map the temporal dynamics of gene expression in the context of cellular differentiation.

The mammary gland is an apocrine gland, and its key function is to produce and secrete milk. The basic components of the adult mammary gland are alveoli, which are lined by milk-secreting epithelial cells. Myoepithelial cells and a stromal compartment that are derived from embryonic ectoderm and mesoderm respectively surround the epithelial cells. Several alveoli join to form lobules that have a lactiferous duct, which drains milk into an opening of the nipple^7^. The development of murine mammary gland initiates during E10-11.5 of embryonic state with the formation of bilateral stripes on either side of the abdomen of a developing embryo, which further organizes into placodes. During puberty, these placodes differentiate into multiple mammary trees and terminal end buds under the influence of estrogen and insulin-like growth factor (IGF)^8^. Further development of mammary gland is triggered at a virgin stage and continues throughout the pregnancy till the end of parturition, under the influence of various hormones including epidermal growth factor (EGF), glucocorticoids (GC) and prolactin (PRL).

The levels of EGF get elevated during early pregnancy^9^, and it is thought to play a critical role in the proliferation of normal epithelium, that is required for the growth of terminal buds of the mammary gland^10^. GC induces genes involved in the maintenance of tight junction complexes and apical junction remodelers such as Adherins, ZO-1 and β-Catenin^11–13^. Furthermore, GC signaling act as a prerequisite for PRL to act on chromatin prepared by GC^14^. However, the specific role of GC in the context of lactogenic differentiation needs to be understood in detail. GC receptor (GR), upon activated by GC, translocate to the nucleus and gets recruited to the glucocorticoid response elements (GRE) to facilitate transactivation of target genes. In general, GR binding sites are located at >2.5 kb upstream of transcription start sites (TSS) of the corresponding genes^14^. Even though GR was shown to interact with GRE, it was not always associated with the expression of corresponding genes. These observations suggest the possibility of involvement in long-range chromatin interactions to elicit GCR/GRE mediated gene expression. On the other hand, PRL was shown to elicit its actions on mammary gland during late pregnancy and lactation. Transcription factors, Stat5a and Stat5b, are the primary inducers of lactogenic gene expression under the stimulus of PRL. Conditional deletion of *STAT5* before and during pregnancy prevents lactogenic differentiation of epithelial cells and also elicits premature cell death, suggesting a critical role of *STAT5* in proliferation, differentiation, and survival of MEC^15^.

Our understanding of the regulation of gene expression during lactogenesis by various hormones has come from the transcriptional regulation studies on a predominant milk protein gene, *β-casein*. Under the stimulus, *β-casein* promoter recruits transcription factors and co-activators at the proximal promoter and enhancers located ~6 kb upstream of its TSS^16^. GC induces the recruitment of p300 at *the β-casein* promoter and enhancer sites leading to acetylation of Histones H3 and H4^16^, which is required for transcriptional activation. PRL signaling promotes recruitment of Hdac1 to the *β-casein* promoter, thereby facilitating transcriptional activation by deacetylation of adjacent enhancer binding protein (CEBP)^16^. Treatment with GC alone did not produce a detectable increase in *β-Casein* mRNA levels. A 3-fold increase in *β-casein* mRNA was detected in cells treated with PRL alone whereas 500-fold induction of β-casein mRNA was observed upon treatment with both GC and PRL^16^. It was also observed that GC treatment alone led to a rapid increase in histone H3 acetylation and treatment with both GC and PRL was required for stable association of p300 and RNA polymerase II at both promoter and enhancer region of *β-casein*, highlighting to the importance of long-range interactions at stage-specific context^16^. Gene expression profiles from multiple mammary cell types representing various developmental stages using 15 K and 20 K oligonucleotide microarray were reported earlier^17–19^. However, these studies do not provide state-specific (Normal, Primed and PRL treated HC11 cells) gene expression/regulatory networks in a comprehensive manner. It is known that mammary gland development occurs at various stages and several hormones including GC and PRL orchestrate these synergistic events. A comprehensive understanding of genome-wide transcriptome profile of MEC representing various stages of mammary gland differentiation would provide greater details of regulation by GC and PRL.

In this study, we comprehensively analyzed the gene regulatory networks of HC11 MEC in response to 1) treatment with EGF and Insulin (considered as normal MEC), 2) Primed with GC (considered as P), and 3) treatment with both GC and PRL (considered as PRL). We also cultured murine embryonic stem cells (ESC) and compared its transcriptome with normal HC11 MEC to identify the dynamics of epithelial cell-specific gene regulatory networks. This study provides an in-depth transcriptome analysis of the profound physiological changes brought about by the action of GC and PRL in MEC.

## Results

### HC11 MEC undergoes G0/G1 arrest during lactogenic differentiation

**Figure 1:**
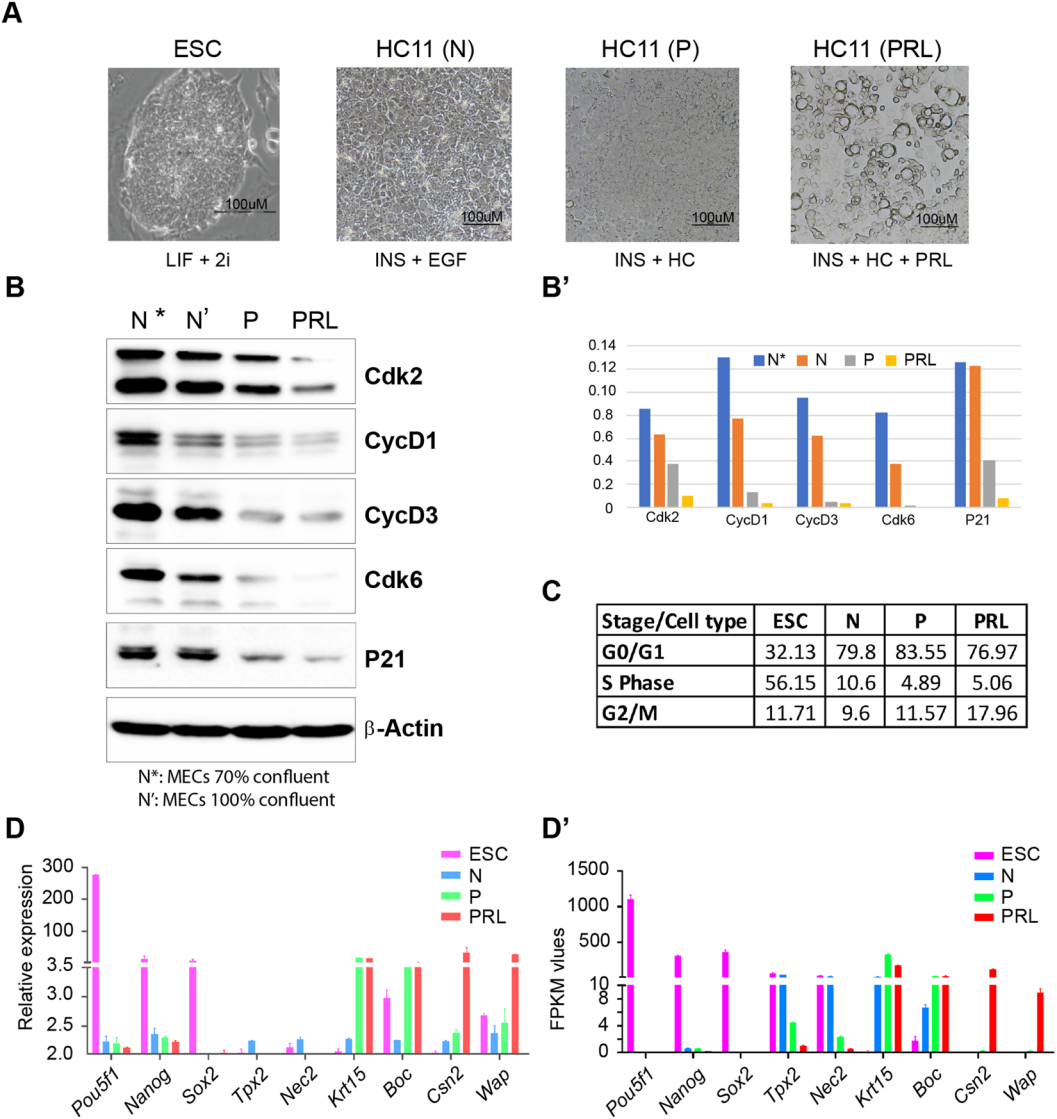
Characterization of HC11 MEC undergoing lactogenic differentiation. (**A**) Bright field microscopic images of actively growing ESC, undifferentiated HC11 cells in presence of EGF and INS (N) and HC11 cells primed with GC (P) alone and HC11 cells treated with GC and PRL. Note the formation of clear dome-shaped mammospheres under PRL condition. Scale bar represents 100 μM. (**B**) Immunoblot analysis of cell cycle regulators in actively growing (N*), confluent stage undifferentiated normal (N’) HC11 cells along with HC primed (P) and PRL treated cells showing a gradual reduction in their levels in comparison with Actin-B. Full-length blot ECL images are provided in Supplementary Fig. S2. (**B’**) Quantitative analysis of cell cycle regulators normalized against β-Actin showing a gradual reduction in their levels during lactogenic differentiation. (**C**) Table showing the percentage of ESC, N, P and PRL treated HC11 cells at G0/G1, S and G2/M phase of cell cycle showing Predominantly in S phase for ESCs and G0/G1 phage for rest of HC11 cell types. (**D**) Real-time PCR analysis of cell-type specific gene expression analysis representing ESC, N, P, and PRL treated HC11 cells. (**D’**) RNA-seq data presentative FPKM values for the respective cell-type-specific genes.

It is known that a regulated withdrawal from the cell cycle allows cells of any kind to differentiate. Therefore, to assess whether lactogenic differentiation of MEC accompanies cell cycle arrest, we performed lactogenic differentiation of MEC as described earlier^20^. We confirmed the formation of mammospheres, which were more prominent after the treatment with PRL (Fig. 1A). Cellular differentiation is associated with cell cycle withdrawal; therefore, we analyzed the expression of cell cycle regulators by immunoblotting. Expression of cyclinD1, D3, CDK2, CDK6, and p21 decreased in differentiated MEC compared to proliferating and confluent normal MEC (Fig. 1B,B’). Cell cycle analyses revealed that majority of cells (77–84%) in MEC (normal & differentiated) were accumulated in the G0/G1 stage, while most of the cells in ESC are in S-phase (Fig. 1C). Accumulation of MEC in G0/G1 stage and expression of cell cycle regulators (Fig. 1B,C) suggests the progression to cell cycle arrest, which may facilitate differentiation. Further, we assessed the expression of specific markers pertinent to stage-specific lactogenic differentiation. ESC expressed the putative markers such as *Pou5f1, Nanog, and Sox2*. HC11 MECs expressed*Tpx2* and *Nek2*, whereas, HC11 primed cells expressed *Krt15* and *Boc* and PRL treated HC11 cells expressed *Csn2* and *Wap* (Fig. 1D). We assessed the expression of specific markers pertinent to lactogenic differentiation based on FPKM values obtained from RNA-seq analysis (See below) and found that identical sets of genes are induced during lactogenic differentiation of HC11 MEC (Fig. 1D’).

### RNA-seq analysis of ESC and differentiated HC11 MEC

To quantify the changes in the expression levels of each transcript during lactogenic differentiation and to comprehensively understand the profile of all the transcripts, we performed RNA-seq and analyzed the data in ESC, normal MEC and MEC treated with GC and PRL. Qualitative analysis of RNA-seq data is provided in the Supplementary Table 1.

**Figure 2:**
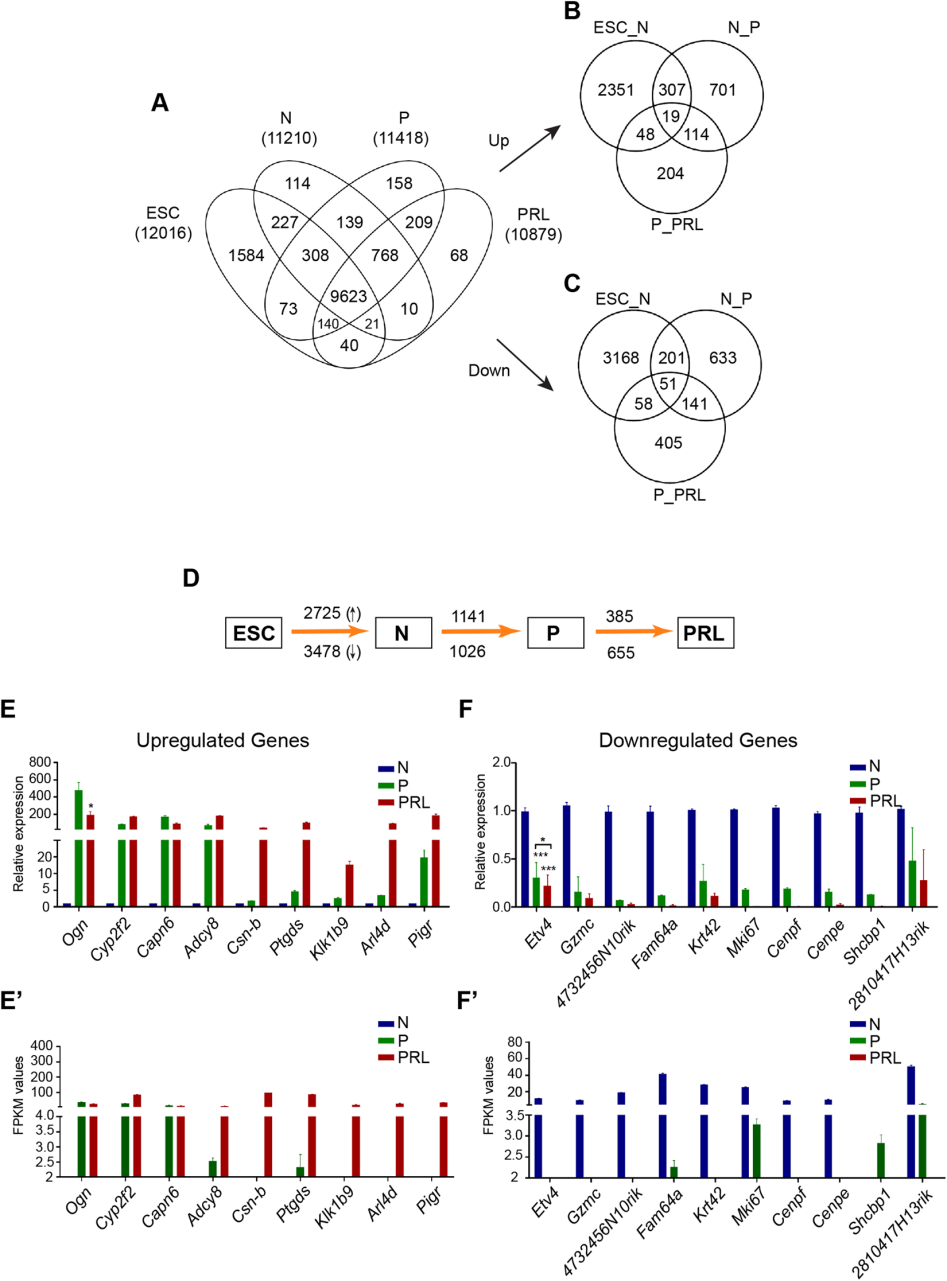
RNA-seq expression analysis of ESC and HC11 MEC undergoing lactogenic differentiation and its validation by real-time PCR. (**A**) Venn diagram showing expression of total, unique and overlapping genes (>1 FPKM value) in ESC, Normal (N), GC primed (P) and PRL treated HC11 cells. (**B**) Venn diagram showing unique and overlapping, upregulated genes between ESC vs N, N vs P and P vs PRL treated HC11 cells (Log2 ≥1). (**C**) Venn diagram showing unique and overlapping, downregulated genes between ESC vs N, N vs P and P vs PRL treated HC11 cells (Log2 ≤−1). (**D**) Schematic diagram showing a total number of genes which are differentially upregulated (Up arrow) and downregulated (Down arrow) between ESC vs Normal (N), N vs GC primed (P) and PRL treated HC11 cells. (**E**) Real-time PCR analysis of the top upregulated and (**F**) downregulated in Normal (N), GC primed (P) and PRL treated HC11 cells and their respective RNA-seq FPKM values of (**E’**) upregulated genes and (**F’**) downregulated genes.

**Figure 3:**
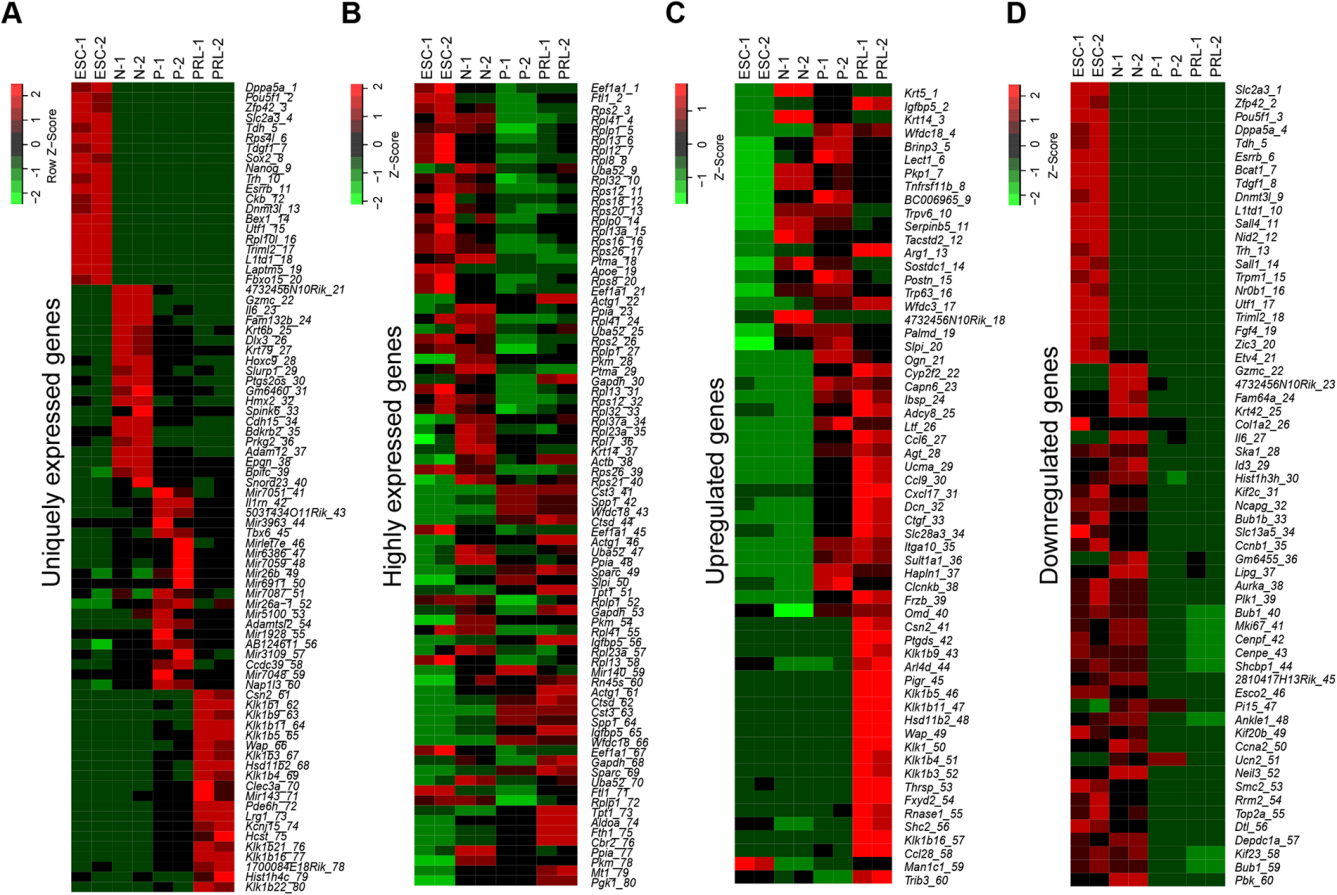
Comparative analysis of unique and differentially expressed genes in ESC and HC11 MEC undergoing lactogenic differentiation. (**A**) Heat map showing top 20 uniquely expressed genes, (**B**) highly expressed, (**C**) up-regulated and (**D**) downregulated genes in replicates of ESC (ESC 1 & 2), Normal HC11 cells (N1 & 2), GC primed (P1 & P2) and PRL treated HC11 cells (PRL1 & PRL 2).

We compared the transcriptome profile of HC11 MEC with ESC after aligning them with GRCm38/mm10 mouse reference genome assembly. In case of ESC, 78% of total reads were uniquely mapped to the reference mouse genome, whereas ~88% of the reads were uniquely mapped in case of both normal MEC and treated MEC (Supplementary Table 2). Mixed datasets of highly correlated (>0.9) duplicate samples (Supplementary Table 3) from ESC, normal, primed and PRL treated MEC were used to derive respective ‘fragments per kilobase per million reads’ (FPKM) values using Sub-read version 1.5.0. We considered genes with an FPKM value of minimum 1, as expressed and found a total of 12016 genes expressed in ESC, 11210 in normal MEC, 11418 in GC treated MEC and 10879 with PRL treated MEC (Fig. 2A; Supplementary Tables 4–7). While a large number of genes (9623) express constitutively in ESC and MEC, relatively less number of genes are differentially expressed during lactogenic differentiation (Fig. 2A). 1584 genes were selectively expressed in ESC, 114 in normal MEC, 158 in primed MEC and 68 in PRL treated MEC (Fig. 2A). It is noteworthy that ESC expressed a large number of unique genes compared with both normal and differentiated MEC (Figs 2A and 3A). We further categorized highly expressed genes from ESC, normal, primed and PRL states based on FPKM values. Among them, genes with higher FPKM values were predominantly housekeeping and ribosomal genes. However, some of these genes were cell-specific, indicating that these might be induced selectively during GC and PRL treatment (Fig. 3B).

### Lactogenic differentiation induces differential expression of a large number of transcripts

We next sought to understand the differentially expressed genes during various stages of lactogenic differentiation. We analyzed differentially expressed genes using DEseq2^21^. A Log2 fold change of ≥+1 and ≤−1 values were considered as a cutoff to define up and downregulation respectively, with a p-adjusted value of <0.001. This analysis showed that 2725, 1141 and 385 genes were upregulated and 3478, 1026 and 655 genes were downregulated when compared ESCs vs. normal, normal vs. GC and GC vs. PRL treated HC11 cells, respectively (Fig. 2B–D; Supplementary Tables 16, 20, 24, 28, 32, 36). It should be noted that the total number of differentially expressed genes (both up and downregulated) were decreased from ESC vs. normal MEC to normal vs. GC treated MEC to GC vs. PRL treated MEC. Although, both GC and PRL are key factors of lactogenic differentiation, a large number of genes were differentially expressed (upregulated: 1141; downregulated: 1026) upon priming with GC than both GC and prolactin treatment (PRL; upregulated: 385 and downregulated: 655). We selected a list of genes such as *Ogn, Cyp2f2, Capn6, Adcy8, Csn2, Ptgds, Klk1b9, Arl4d* and *Pigr* (upregulated), and *Etv4, Gzmc, 4732456N10rik, Fam64a, Krt42, Mki67, Cenpf, Cenpe, Shcbp1* and *2810417H13Rik* (downregulated) based on RNA-seq data from all three types of MEC and validated their expression by RT-PCR (Fig. 2E,F). Expression of each transcript by RT-PCR is in concurrence with respective FPKM values (Fig. 2E’,F’). Top 20 uniquely expressed genes in ESC, N: Normal P: Primed and Prolactin treated MEC are represented as a heat map (Fig. 3A). Similarly, top 20 highly expressed genes (Fig. 3B), top 20 highly upregulated genes (Fig. 3C) and top 20 highly downregulated (Fig. 3D) genes between ESC, N, P and PRL treated MEC were represented as heat maps.

### Lactogenic differentiation induces differential expression of a large number of Transcription factors and epigenetic regulators

**Figure 4:**
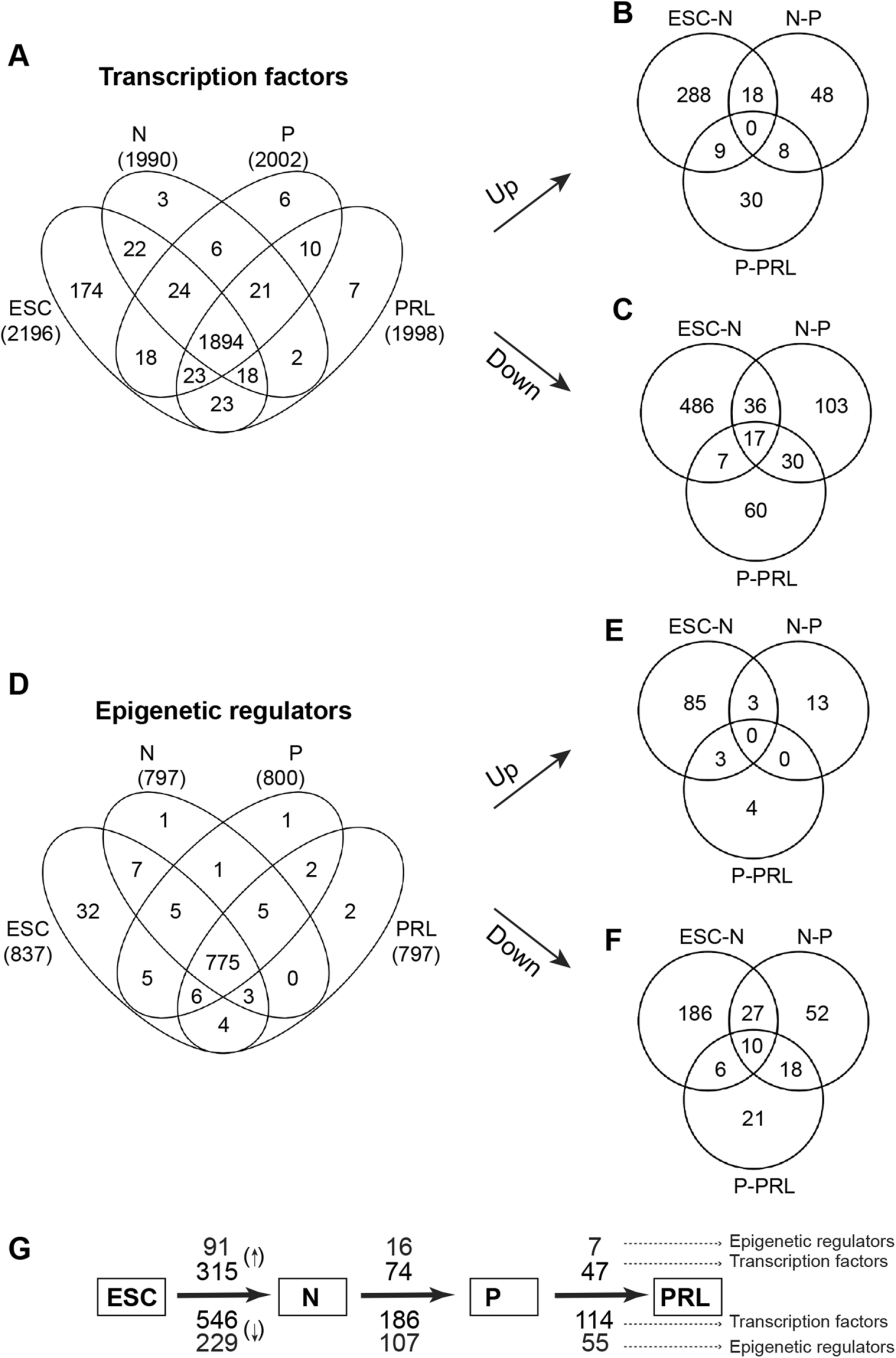
RNA-seq expression analysis of transcription factors (TFs) and epigenetic regulators (ERs) in ESC and HC11 MEC undergoing lactogenic differentiation. (**A**) Venn diagram showing total, unique and overlapping expression of TFs between ESC vs N, N vs P and P vs PRL treated HC11 cells (>0 FPKM). (**B**) Venn diagram showing unique and overlapping expression of upregulated (log2 ≥1) and (**C**) downregulated (≤−1 FPKM) TFs between ESC vs N, N vs P and P vs PRL treated HC11 cells. (**D**) Venn diagram showing unique and overlapping expression of ERs between ESC vs N, N vs P and P vs PRL treated HC11 cells (>0 FPKM). (**E**) Venn diagram showing unique and overlapping expression of upregulated (Log2 ≥1) and (**F**) down-regulated (Log2 ≤−1) ERs between ESC vs N, N vs P and P vs PRL treated HC11 cells. (**G**) Schematic diagram showing a total number of TFs/ERs which are differentially upregulated (Up arrow) and downregulated (Down arrow) between ESC vs Normal (N), N vs GC primed (P) and PRL treated HC11 cells.

**Figure 5:**
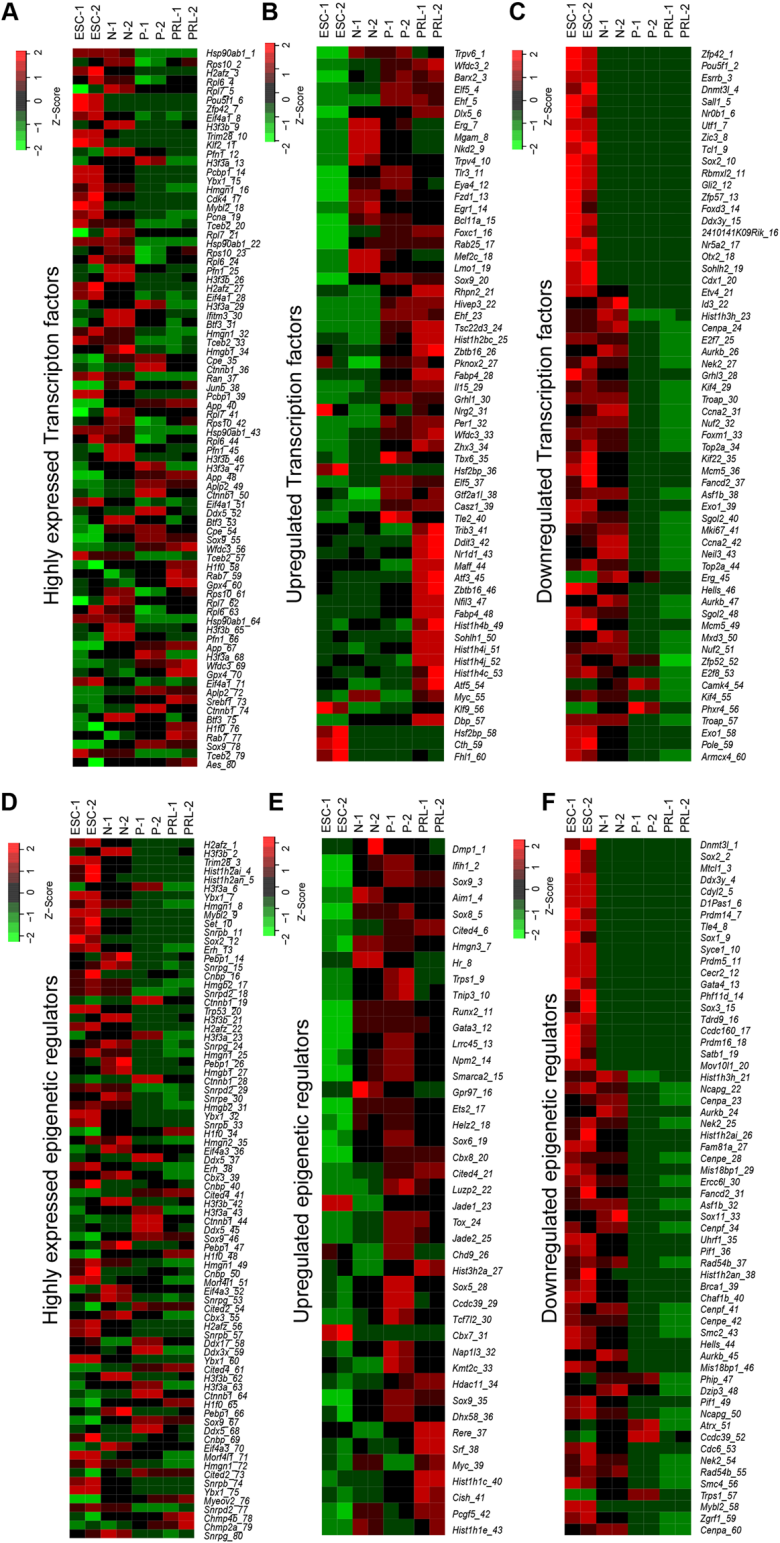
Comparative analysis of unique and differentially expressed TFs and ERs in ESC and HC11 MEC undergoing lactogenic differentiation. (**A**,**B** and **C**) Heat map showing top 20 highly expressed, up-regulated and down-regulated TFs and (**D**,**E** and **F**) ERs respectively in replicates of ESC (ESC 1 & 2), Normal HC11 cells (N1 & N2), GC primed (P1 & P2) and PRL treated HC11 cells (PRL1 & PRL 2).

It is generally considered that the expression of cell-type-specific genes is under the control of TFs^3,4^ and ERs^5^. Therefore, we analyzed TFs and ERs that were differentially transcribed during lactogenic differentiation of MEC (Supplementary Tables 8–15). We observed that 2196, 1990, 2002 and 1998 TFs are expressed in ESC, normal, primed and PRL states of MEC, respectively (Fig. 4A). On the other hand, 837, 797, 800 and 797 ERs were expressed in ESC, normal, primed and PRL states of MEC, respectively (Fig. 4D). It is noteworthy that 1894 TFs and 775 ERs were constitutively expressed in both ESC and MEC (Fig. 4A,D). Next, we compared the expression of these regulatory factors during various stages of lactogenic differentiation. We observed that 315, 74 and 47 TFs (Fig. 4B) and 91, 16 and 7 ERs (Fig. 4E) were upregulated in ESC vs. normal MEC, normal vs. GC primed, and primed vs. PRL treated MEC, respectively. Similarly, we found that 546, 186 and 114 TFs (Fig. 4C), and 229, 107 and 55 ERs (Fig. 4F) were downregulated in ESC vs. normal, normal vs. GC primed and primed vs. PRL treated MEC, respectively. Although a large number of TFs and ERs are differentially expressed between ESC and MEC, priming of MEC with GC resulted in more differentially expressed TFs and ERs than PRL treatment. The total number of up and downregulated TFs and ERs during lactogenic differentiation of MEC are summarized in Fig. 4G. Heatmaps for highly expressed, up-and downregulated TFs are provided in Fig. 5A–C. Heatmaps for highly expressed, up-and downregulated ERs are provided in Fig. 5D–F.

### Analysis of gene expression between ESC vs. normal HC11 MEC

#### Upregulated genes

**Figure 6:**
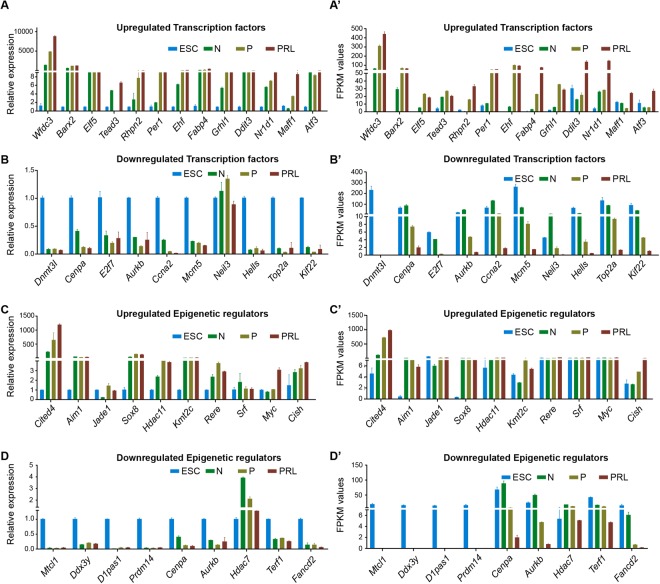
Real-time PCR validation of top up or downregulated TFs and ERs in ESC and HC11 MEC undergoing lactogenic differentiation. (**A**) Bar chart showing real-time PCR measured relative expression of upregulated and (**B**) downregulated TFs along with its respective RNA-seq FPKM values (**A’** and **B’**) in ESC, N, P and PRL treated HC11 cells. (**C**) Bar chart showing real-time PCR measured relative expression of up-regulated and (**D**) down-regulated ERs along with its respective RNA-seq FPKM values (**C’** and **D’**) in ESC, N, P and PRL treated HC11 cells.

**Figure 7:**
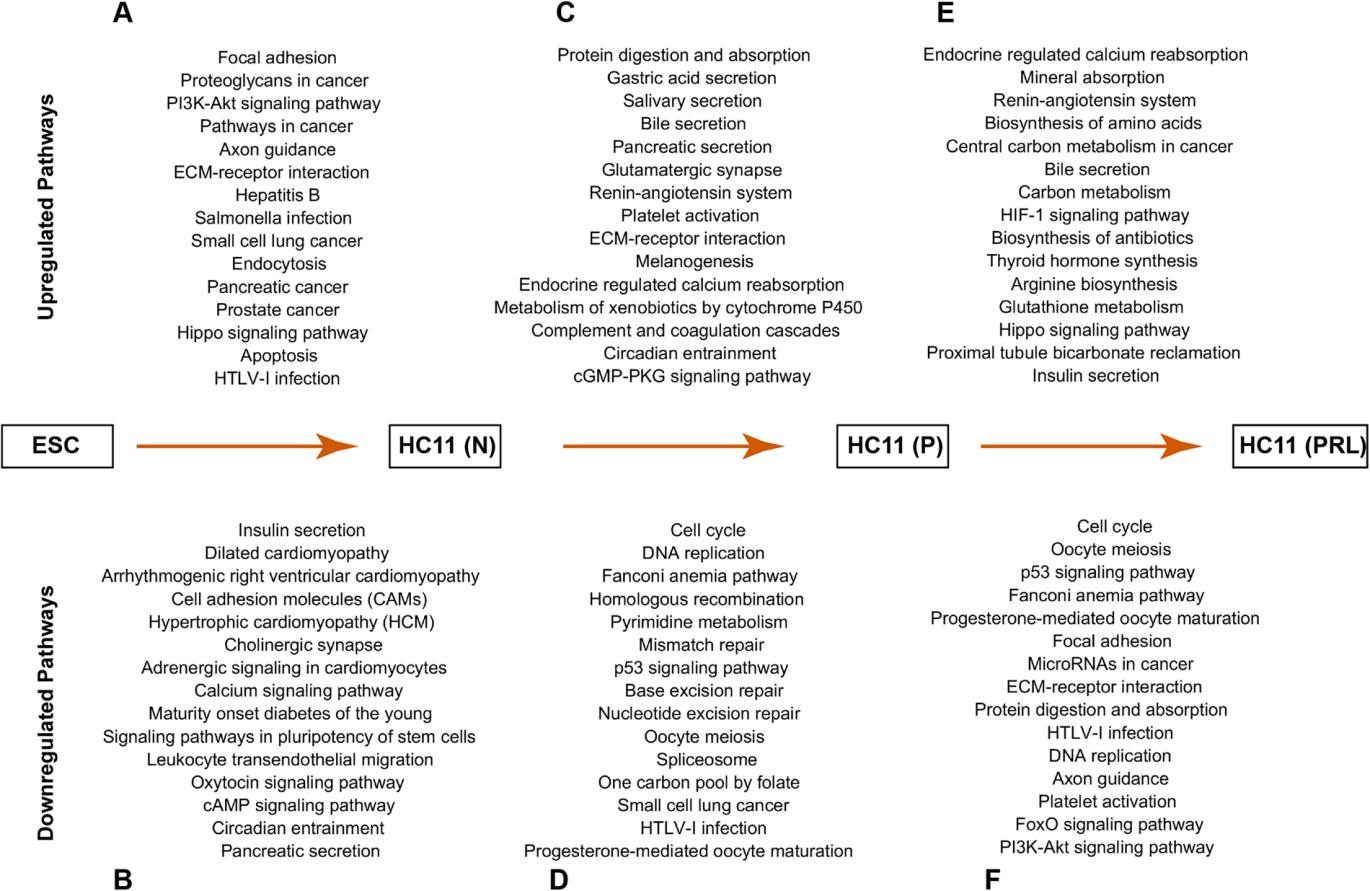
Pathway analysis of genes that are differentially expressed between ESC and HC11 MEC undergoing lactogenic differentiation. (**A**, **C** & **E**) KEGG pathways analysis of genes that are either upregulated and (**B**,**D** & **F**) downregulated between ESC vs normal HC11 cells (N), N vs GC primed (P) and P vs PRL treated HC11 cells respectively.

A total of 2725 genes were significantly upregulated in normal MEC when compared with ESC (Fig. 2D). Among them, *krt5, Igfbp5, Krt14*, and *Brinp3* were significantly upregulated (Fig. 3C; Supplementary Table 16). These genes encode for keratins, that are involved in extracellular matrix support, and IGF binding protein, which helps in the proliferation and survival of MECs^22^. We also found that 315 TFs and 91 ERs were significantly upregulated in normal MEC compared to ESC (Fig. 4G; Supplementary Tables 17 and 18). *Trpv6, Wfdc3, Barx2, Elf5* and *Ehf* (TFs), and *Dmp1*, *Ifih1, Sox9, Aim1* and *Sox8* (ERs) were significantly upregulated in normal MEC (Fig. 5B,E). We confirmed the expression of top upregulated TFs (Fig. 6A) and ERs (Fig. 6C) by RT-PCR. FPKM data of RNA-seq for these TFs and ERs is comparable to that of RT-PCR data (Fig. 6A’,C’). Among TFs that are upregulated, *Elf5* is very specific to normal MEC and shown to regulate mammary stem or progenitor cell population^23,24^. Based on upregulated genes, we performed KEGG pathway analysis and found several pathways activated in MECs including focal adhesion, proteoglycans and PI3k-Akt signaling pathway (Fig. 7A; and Supplementary Table 19). We also performed different pathway analyses like BioCarta, and Wiki pathways, which are shown in Supplementary Fig. S1 and pathway scores, are shown in Supplementary Tables 40 and 41.

#### Downregulated genes

Similarly, we observed that about 3478 genes were downregulated in normal MEC compared to ESC (Fig. 2D; Supplementary Table 20). The downregulated genes include *Nanog, Apoe, Akap12, Mybl2*, and *Gm2373* (Fig. 3D). Most of the genes that were downregulated found to be associated with the maintenance of stem cell state. We identified that 546 and 229 TFs and ERs are downregulated in MEC, respectively (Fig. 4G; Supplementary Tables 21 and 22). *Zfp42, Pou5f1, Esrrb*, and *Dnmt3l* are among the top downregulated TFs (Fig. 5C), whereas *Sox2, Mtcl1, Ddx3y*, and *Cdyl2* are the top downregulated ERs (Fig. 5F). Among them, *Pou5f1 and Sox2* are very important for the maintenance of ESC pluripotency^25,26^. The decreased expression of these factors in MEC suggests that these cells are differentiated. We confirmed the expression of top downregulated TFs (Fig. 6B) and ERs (Fig. 6D) by RT-PCR. FPKM data obtained from RNA-seq for these TFs and ERs is comparable to that of RT-PCR data (Fig. 6B’,D’). KEGG pathway analysis showed that genes involved in insulin secretion, dilated cardiomyopathy, arrhythmogenic right ventricular cardiomyopathy (ARVC), cell adhesion molecules and hypertrophic cardiomyopathy (HCM) were downregulated in MEC compared to ESC (Fig. 7B; Supplementary Table 23).

### Analysis of gene expression in HC11 MEC during GC priming

#### Upregulated genes

A total of 1141 genes were differentially expressed in MEC treated with GC compared to normal MEC (Fig. 2D). Some of the upregulated genes include *Ogn, Cyp2f2, Capn6, Ibsp*, and *Adcy8* (Fig. 3C; Supplementary Table 24). Interestingly, we found only 74 TFs and 16 ERs upregulated in GC primed vs normal MEC (Fig. 4G; Supplementary Tables 25 and 26). Upregulated TFs in GC primed MEC include *Rhpn2, Hivep3, Ehf* and *Tsc22d3* whereas upregulated ERs include *Cited4, Luzp2, Jade1* and *Tox* (Fig. 5B,E). We have validated the expression of significantly upregulated TFs and ERs by RT-PCR (Fig. 6A,C). The expression of upregulated TFs and ERs is in concurrence with FPKM data of RNA-seq (Fig. 6A’,C’). The epigenetic regulator and a transcriptional coactivator, *Cited4* that was upregulated during priming is known to be involved in milk secretion from mammary gland^27^. Further, genes shown to play a role in chromatin remodeling and decompaction such as *Chd9*^28^ and *Myc*^29^ are significantly upregulated during priming with GC (Figs 5E, 6C and C’). Most of the upregulated genes are known to be involved in pathways corresponding to protein digestion and absorption, gastric acid secretion, salivary secretion, glutamatergic synapse, melanogenesis and circadian entrainment (Fig. 7C and Supplementary Table 27).

#### Downregulated genes

We found a total of about 1026 genes that were significantly downregulated in HC11 MEC during priming with GC (Fig. 2D; Supplementary Table 28), which include *Etv4, Gzmc, 4732456N10rik, Fam64a* and *Krt42* (Fig. 3D). Moreover, we found that 186 TFs and 107 ERs were downregulated in GC primed HC11 MEC (Fig. 4G; Supplementary Tables 29 and 30). *Etv4, Id3, Hist1h3h, Cenpa*, and *E2f7* are some of TFs that were downregulated during priming (Fig. 5C), whereas *Ncapg, Aurkb, Nek2, Hist1h2ai*, and *Cenpe* are some of the downregulated ERs (Fig. 5F). The TFs *Etv4, E2f7*, and *Aurkb* are known to be involved in cellular proliferation^30–32^. Further, we validated the expression of top downregulated TFs (Fig. 6B) and ERs (Fig. 6D) by RT-PCR and decreased expression of these TFs and ERs during priming is comparable with FPKM data obtained from RNA-seq (Fig. 6B’,D’). The significantly downregulated genes were mostly involved in the cellular proliferation, DNA binding, and cellular growth-related functions. Pathway analysis of the downregulated genes showed that pathways related to cell cycle, DNA replication, Fanconi anemia pathway, homologous recombination as well as pyrimidine metabolism are all downregulated (Fig. 7D; Supplementary Table 31).

### Analysis of gene expression in PRL treated HC11 MEC

#### Upregulated genes

To gain further insight into the changes in HC11 MEC during lactogenic differentiation, gene expression profile, pathway analysis and expression of TFs and ERs were analyzed in GC treated vs. prolactin + GC (PRL) treated HC11 MEC. Gene expression analysis revealed a total of 385 genes to be significantly upregulated in MEC treated with PRL vs. MEC treated with GC (Fig. 2D; Supplementary Table 32). *Csn2, Wap, Pip, Ptgds, Klk1b9, Arl4d*, and *Pigr* are some of the highly expressed genes in MEC treated with PRL (Fig. 3A,C). It is interesting to note that PRL triggers the expression of major milk proteins such as *Csn2*, *Wap*, and *Pip*, which constitute up to 80% of total milk proteins. We noticed that 47 TFs and 7 ERs were upregulated with PRL treatment (Fig. 4G; Supplementary Tables 33 and 34). *Trib3, Ddit3, Nr1d1, Maff*, and *Atf3* are some of the TFs upregulated with PRL treatment and *Rere, Srf* and *Myc* are some of the ERs highly expressed in PRL treated HC11 MECs (Fig. 5B,E). Upon validation of top upregulated TFs (Fig. 6A) and ERs (Fig. 6C) by RT-PCR, it was revealed that the RT-PCR confirms the FPKM data (Fig. 6A’,C’). *Trib3*, a TF that is associated with various ER stress responses, is upregulated in the lactating period, which requires a high demand for protein synthesis^33^. Xbp1 is another TF that was upregulated during PRL treatment and is required for proliferation and differentiation of MEC^34^. KEGG analysis revealed that PRL activates several other pathways that include endocrine and other factor-regulated calcium reabsorption, mineral absorption, renin-angiotensin system and biosynthesis of amino acids (Fig. 7E and Supplementary Table 35).

#### Downregulated genes

In PRL treated HC11 MEC, 655 genes were downregulated compared with primed MECs (Fig. 2D). Genes such as *Mki67, Cenpf, Cenpe*, and *Shcbp1* were downregulated upon PRL treatment (Fig. 3D; Supplementary Table 36). We found that 114 TFs and 46 ERs, respectively are downregulated in PRL treated MEC (Fig. 4G; Supplementary Tables 37 and 38). Highly downregulated TFs are *Mki67, Ccna2, Neil3, Top2a* and *Erg*, and ERs are *Cenpf, Cenpe, Smc2, Hells* and *Aurkb* (Fig. 5C,F). We validated the expression of downregulated TFs and ERs by RT-PCR (Fig. 6B,D) and is comparable with FPKM data (Fig. 6B’,D’). KEGG analysis for downregulated genes revealed several pathways that are affected such as cell cycle, p53 signaling, Fanconi anemia pathway, and focal adhesion (Fig. 7F; Supplementary Table 39).

### Confirmation of specific factors expression during lactogenic differentiation

**Figure 8:**
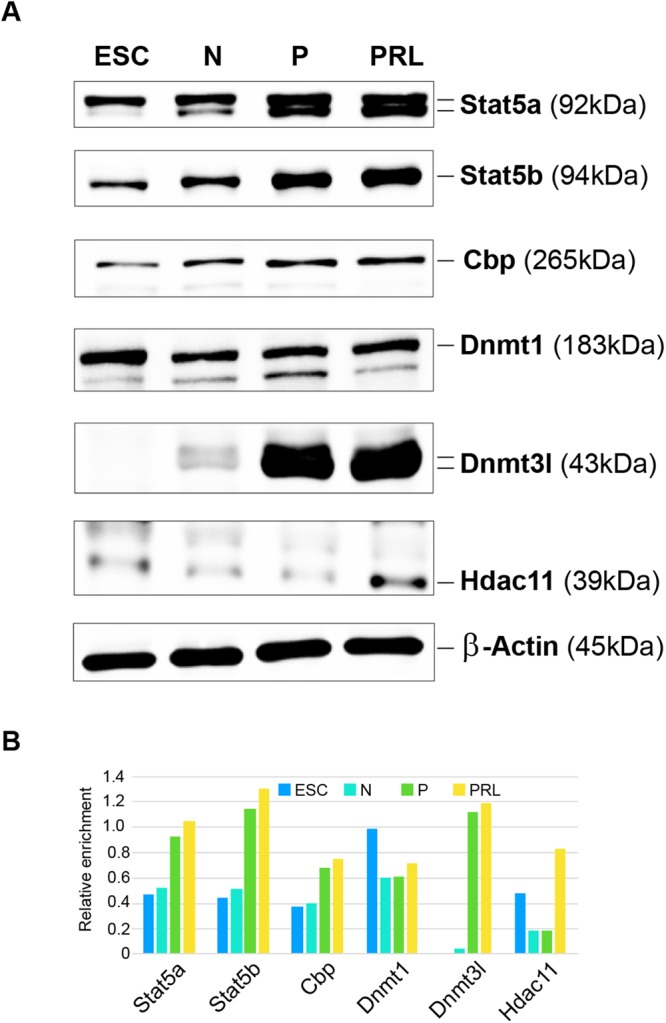
Validation of selective markers in ESC and HC11 MEC undergoing lactogenic differentiation. (**A**) Immunoblots showing steady-state levels of selective markers in ESC, N, P, and PRL treated HC11 cells. (**B**) Bar chart showing relative abundance of respective protein normalized against β-actin levels.

In addition to mRNA expression analysis, immunoblotting for selected markers were performed to determine their expression during lactogenic differentiation of HC11 MECs (Fig. 8A,B). Expression of Stat5a and 5b were increased during differentiation and mainly, the expression of Stat saturated during priming. Interestingly, expression of Dnmt3l protein level is increased during the course of differentiation of HC11 MECs in contrast to RT-PCR data and FRKM data (Fig. 6B,B’). Furthermore, our data reveal that Hdac11 expression increased with PRL treatment (Fig. 8A,B).

## Discussion

The development of mammary gland and lactogenic differentiation of MEC are highly complex biological processes and mediated by several endocrine factors including glucocorticoids, prolactin, insulin, and EGF. Precise and coordinated control of gene expression is vital for lactogenesis. Recent microarray-based studies in HC11 MECs investigated the role of PRL and its relevance to breast cancer^18^. However, the mechanism of multistage lactogenic differentiation of MEC remains unclear. To gain insights into the mechanism of lactogenic differentiation, we employed HC11 MEC and compared the profile of transcripts from normal, GC primed, and PRL treated cells. Further, we garnered the clues about key TFs and ERs that drive the lineage-specific differentiation of ESC to MEC. It is interesting to note that priming induces PRL receptor in MEC, which could help in eliciting PRL signaling following treatment with PRL. Our data suggest that lactogenic differentiation of MEC occurs in a hierarchical manner, where priming with GC might alter chromatin accessibility and sets a base required for the PRL to elicit its action during lactation.

HC11 MEC are widely used in lactogenic differentiation system *in vitro* and form mammospheres during lactogenic differentiation^20^. The transcriptome profile revealed that only a subset (22%) of genes was differentially expressed in MEC when compared to ESCs. *Sox9, Runx2, Gata3, Ehf, Elf5, a*nd *Bcl11a* are TFs and ERs that are critical for the mammary gland development and are reported to maintain luminal progenitor population in the mammary gland^35–38^. We found that these factors are highly expressed in normal HC11, which resembles luminal progenitor cells of the mammary gland. We found up-regulation of Smad3 in MEC when compared to ESC. Smad3 is a downstream molecule of TGF-β pathway and antagonizes STAT5/PRL signaling and promotes cellular proliferation of normal mammary epithelial cells^39^. We also observed that major regulators of stemness such as *Pou5f1, Sox2*, and *Nanog* are downregulated in MEC indicating that cell type-specific gene expression is prerequisite for specialized functions.

We observed that some of the genes (*Brca1, Krt6a, Krt6b, Krt5*, and *Melk)*, which are indicative of stem cell-like characteristics are highly expressed in normal MEC, and are downregulated during differentiation, are majorly involved in cell proliferation. We speculate that normal HC11 cells become proliferative upon exposure to EGF. *Ncapg, Cenpa, Cenpe, Arukb, Kif4*, and *Kif22* are some of the genes involved in mitotic chromosome condensation and segregation and are majorly required for cellular proliferation. These genes are upregulated in normal MEC whereas, in response to GC treatment, these genes are downregulated. Another class of Ets family of TFs are *Etv4* and *Etv5* which are majorly involved in cell motility and cell growth are significantly downregulated since they are not required for normal MEC^40^. During priming with GC, HC11 MEC ceases cell proliferation concomitant with downregulation of above-mentioned genes.

Further, our data suggest GC alone brings about differential expression of a vast number of genes (Fig. 2). Priming of MECs with GC induces various ERs; *Cited4, Luzp2, Jade1, Jade2, Chd9, Myc*, and *Tox*. Earlier studies showed that *Cited4* is actively involved during lactogenic differentiation^27^. *Cited4* binds to CBP/p300, a transcriptional regulator as well as isoforms of the *TFAP2* transcription factor. Cited4 thereby modulates *TFAP2* dependent genes, thus might be helpful to access the chromatin by signal transducers and transcriptional activators during PRL treatment^27,41–43^. Jade family proteins serve as transcriptional activators via their histone acetyltransferase activity^44^ and *Id* family TFs are diversely expressed during mammary gland development. *Id2*, which gets induced during GC treatment, is crucial for survival and differentiation of MEC. This suggests that GC play a critical role to ensure differentiation of MEC during lactogenesis^45,46^. GC activated GCR was shown to remodel chromatin in Brg1 dependent and independent manner^47^. In this regard, GC induction of Chd9, a remodeling enzyme shown to play a role in loosening chromatin in growing oocyte^28^ and Myc, a protein shown to play an essential role in decompaction of chromatin^29^ suggest that there might be a link between these factors in global remodeling of chromatin, however, its relevance in the context of GC signaling remains to be explored further.

Transcriptome profile of HC11 MEC treated with PRL revealed that only 3.5% of genes were differentially expressed compared to GC primed HC11 MEC. Out of the highly expressed TFs and ERs, *c-Myc* is expressed during lactation. Knockdown studies of *c-Myc* in mammary gland showed a defect in alveolar cell proliferation and differentiation resulting in decreased milk production^48^. High expression of E-Cadherin (*Cdh1*) and down-regulation of *Klf4* in HC11 cell-types suggest that HC11 cells are partial primary cells^49^ as it was known that Klf4 suppresses epithelial to mesenchymal transition in breast cancer cells through induction of E-cadherin. PRL treatment induces milk fatty acid biosynthesis by inducing expression of various TFs such as *Fabp4, Lipe*, and *Srebf1*, which are primary regulators of fatty acid homeostasis in lactating mammary gland^50–52^. In this context, we observed PRL mediated actions are preferably localized to milk production and survival of lactating epithelial cells, as GC prepares these cells for PRL by modulating the accessibility of chromatin and survival of epithelial cells.

Previously reported transcriptome data of HC11 cells was obtained using microarray and are of 15 and 20 K gene probe resolution^17,19^. In this study, we provided RNA-seq of higher resolution with nearly 50–60 million reads, whereas the recommended coverage is 15–20 million read^53^. The data in our study provides greater insight into gene expression in response to GC and PRL treatment during lactogenic differentiation of HC11 mammary epithelial cells.

## Materials and Methods

### Cell culture and lactogenic differentiation of HC11 MEC

HC11 MEC were cultured and differentiated essentially as described earlier^20^ Briefly, murine HC11 MEC stem-like cell line with passage number 6 (from Dr. Nancy E Hynes laboratory, Switzerland) were cultured with RPMI1640-GlutaMAX^TM^ (Gibco) medium supplemented with 10% FBS (US Origin GIBCO), insulin (5 µg/ml, Sigma Catno#I6634), 1X Anti-biotic/Anti-mycotic (Gibco), and EGF (20 ng/ml, Sigma Cat.no#E4127) until they reach confluence under 5% CO_2_. Lactogenic differentiation of HC11 cells was initiated by withdrawal of EGF containing medium and replacement with fresh priming medium consisting of RPMI1640-GlutaMAX^TM^ complete medium with 5% FBS, insulin (5 µg/ml), hydrocortisone (1 µg/ml, Sigma #H4001) and were grown at 37 °C for 48 hrs under 5% CO_2_. Alternatively, the primed cells were subjected to PRL treatment by supplementing with fresh RPMI1640-GlutaMAX^TM^ complete medium with 5% FBS, insulin (5 µg/ml), hydrocortisone (1 µg/ml) and prolactin (5 µg/ml, NIH, #NIDDK-oPRL-21) and cells were then incubated at 37 °C for 72 hrs under 5% CO_2_ to complete lactogenic differentiation. Cells were harvested at 72 hrs and referred to them as PRL state of HC11 MEC.

### Culturing of murine embryonic stem cells ESC

The mouse ESC line R1 was maintained on a layer of mitomycin-C treated mouse embryonic fibroblast feeder cells and harvested as described^54^ with few modifications. Briefly, mouse embryonic fibroblasts were grown at ~60% confluence in a 0.1% gelatin-coated Petri plates with a standard DMEM medium. Fibroblasts were inactivated by replacing standard medium with DMEM containing 5% FBS and 2.5% mitomycin-C for 3 hrs. R1 ESC were revived on inactivated fibroblasts using modified DMEM medium at 37 °C and 7% CO_2_. The components of modified DMEM include 10% FBS, mouse leukemia inhibitory factor (LIF) (1000 U/ml), inhibitors (MEK 0.5 µg/ml: GSK: 1.5 µg/ml), 1% Penicillin-streptomycin solution, 1X Non-essential amino acids, 2mM L-glutamine, and 50 μM 2-Mercaptoethanol. Grown Ebs of ESC on fibroblast feeder cells were subjected to trypsinization (0.5% Trypsin for 30 sec at 37 °C) and individual ESC that were devoid of fibroblasts were transferred onto fresh Petri plates and cultured in DMEM medium containing 10% FBS. ESC were selectively enriched over fibroblasts owing to the potential of fibroblasts to attach to the Petri plates faster than ESCs. These ESC were harvested for total RNA isolation, RNA-seq library preparation and sequencing.

### Cell cycle analysis

Cell cycle analysis was performed for ESC, normal HC11 cells, HC11 cells exposed to priming conditions, and PRL treatment. Respective cells were cultured as detailed above, trypsinized and were washed three times with PBS (0.1 M, pH 7.4). Following washing with PBS, cells were fixed in 70% ethanol at −20 °C for 24 h. After fixation, cells were washed with ice-cold PBS and stained with buffer containing propidium iodide (50 μg/ml) and RNaseA (200 mg/ml), at 37 °C for 30 min in the dark. Cells cycle analysis was performed using the flow cytometer (BD Fortessa).

### RNA extraction, library construction, and sequencing

Total RNA from ESC, Normal MEC, GC primed, and PRL treated MEC was isolated using Trizol (Invitrogen#15596026) according to manufacturer instructions. RNA was further purified using a commercially available kit (GCC Biotech#GR1003). 20 μg of purified RNA from each sample was treated with 10 Units of DNase1 (Roche # 04716728001) and were further purified by using G Sure cell culture RNA isolation kit. From each RNA sample, Ribosomal RNA was removed using Ribo-Zero kit (NEB#E6310L), and further mRNAs were enriched using Oligo (dT) beads. Illumina paired-end library was prepared as per the NEBNext® Ultra™ RNA Library Prep Kit (NEB#E7530S). All the libraries were paired-end sequenced using Illumina HiSeq2500 sequencing platform.

### RNA-seq reads quality control and mapping

Raw sequence reads in the FASTQ format were further processed to remove Illumina adaptor sequences by using Trimmomatic^55^. The resultant raw reads were compressed to.gz format and were deposited in GEO repository (GSE107419). After filtering, approximately 55–65 million paired-end reads were processed for each sample. The reads were mapped to mouse reference genome (GRCm38/mm^10^) using TopHat2.1.0^45^. The mismatch parameter was set to 2 and all other parameters to default.

### Quantification and Identification of differentially expressed genes

Quantification of the expression of different genes was done with the tool ‘Sub-read version1.5.0’ using the features count mode method. Briefly, Total read count for each gene was obtained and was then normalized to obtain FPKM (Fragments per Kilobase exon per million reads sequenced) values. An FPKM ≥ 1 was considered as a threshold for determining whether a gene is expressed or not^56^. For differential gene expression analysis, DESeq2 version 1.24 was used. Differential gene expression analysis involved steps like model dependent p-value estimation using negative binomial distribution and Wald test, and adjusted p-value estimation based on multiple hypothesis testing. A log2 Fold change value of ≥1 is taken as a cutoff to define upregulated and a value of ≤−1 to define downregulated genes. The p-adjusted threshold value was set to 0.001 to maintain statistical significance. gg-plots package (R programming) was used to generate the heat maps representing the data. Venn diagrams representing the data were generated using Ugent web tool (http://bioinformatics.psb.ugent.be/webtools/Venn/).

### Identification of differentially expressed TFs and ERs

TFs in mouse were identified using the sequence-specific DNA binding or transcription regulation terms in GO functional annotation^57^ (Supplementary Table 44). List of ERs and their coding genes were obtained from previous literature^58–60^ (Supplementary Table 45). Corresponding expression (>0 FPKM) and log2 fold change values (≥1 up-regulation; ≤−1 downregulation) of TFs and ERs were extracted from the RNA-seq data. Here, it should be, however, note that some of the genes are represented both under TFs and ERs (Supplementary Table 46). Heat maps and Venn diagrams were generated as described in the previous section.

### Pathway analysis of differentially expressed genes

Top upregulated and downregulated genes in ESC vs normal, normal vs Primed and Primed vs PRL were uploaded to DAVID database^61,62^ to derive the KEGG pathway enrichment and GO annotation data. All differentially expressed gene lists were uploaded in DAVID functional annotation from which KEGG pathway enrichment scores list was derived. Biocarta and Wiki pathways were obtained from existing databases^63,64^. Gene set enrichment analysis was calculated similarly to the method published previously^65,66^, and log2 fold change of each gene was calculated by comparing different samples. Mean log2 fold change of a pathway was calculated by summing the scores of individual genes. Gene-set enrichment score was calibrated against the background distribution using randomly sampled n (number of genes in a pathway) scores and calculating mean log2 score. This process was repeated over 10000 times. The mean and standard deviation of the sampling distribution thus obtained was used in correcting the original score.

### Real-time RT-PCR analysis

Differentially expressed genes in RNA-seq analysis were validated by quantitative real-time PCR (CFX96#Bio-Rad). DNase treated 1 µg RNA was taken as input from each sample to synthesize cDNA using iScript cDNA synthesis kit (Bio-Rad). cDNA template was diluted, and PCR conditions were followed according to manufacturer protocol (Kappa Biosystems). *β-actin* was used as a housekeeping internal control gene and controls with no template were also included in PCR plate. All primers used in this experiment were designed to span exon-exon junctions to minimize genomic DNA contamination. Relative gene expression was calculated using the method −2^ΔΔCT ^^67^. All the graphs were generated using Graph pad prism software. Significance was calculated using two-way ANOVA by using graph pad prism and represented as [*] on top of error bars. All gene-specific primers used were listed in Supplementary Table 42.

### Immunoblotting: Cell lysate from ESC and MEC (normal, Primed and Prolactin treated) was prepared and protein was extracted using RIPA buffer

A total of 30 μg of protein from each sample was resolved on 8% SDS-PAGE and subsequently transferred to nitrocellulose membrane by electroblotting. The nitrocellulose membrane was blocked in 5% non-fat milk for 1 hr and cut as per the respective protein molecular weight range. Subsequently, all the blots were incubated with the respective primary antibody at 4 °C overnight and then incubated with appropriate HRP linked secondary antibodies for 1 hr at room temperature. Blots were developed using the enhanced chemiluminescence (Bio-Rad#1705062) and imaged using Chemidoc (Bio-Rad). The protein band intensities were quantified using Image J software and normalized to β-actin expression. All the raw and contrast adjusted blots are represented in the supplementary information (Supplementary Fig. S2). All the antibodies and dilution factors used for this study were listed in Supplementary Table 43.

### Data availability

All the raw data generated and analyzed in this study were deposited in GEO, accession number: GSE107419.

## Electronic supplementary material

Supplementary Information

Supplementary Table
