# Supplementary material for: Comprehensive profiling of transcriptional networks specific for lactogenic differentiation of HC11 mammary epithelial stem-like cells
Source: Sci Rep. 2018 Aug 6;8:11777. doi: 10.1038/s41598-018-30122-4 (PMC6079013; doi:10.1038/s41598-018-30122-4)
Supplement: Supplementary file 1 — Supplementary Information [file 41598_2018_30122_MOESM1_ESM.pdf]

Comprehensive profiling of transcriptional networks specific for lactogenic differentiation of mammary epithelial stem-like cells

Trinadha Rao Sornapudi[1], Rakhee Nayak[1], Prashanth Kumar Guthikonda[1], Anil Kumar Pasupulati[3], Srinivas Kethavath[1], Vanita Uppada[1], Sukalpa Mondal[1], Sailu Yellaboina[2], and Sreenivasulu Kurukuti[1*]

1. Department of Animal Biology, School of Life Sciences, University of Hyderabad, Hyderabad-500046, India.
2. CR Rao Advanced Institute of Mathematics, statistics and computer sciences, University of Hyderabad campus, Gachibowli, Hyderabad-500046, India
3. Department of Biochemistry, School of Life Sciences, University of Hyderabad, Hyderabad-500046, India.

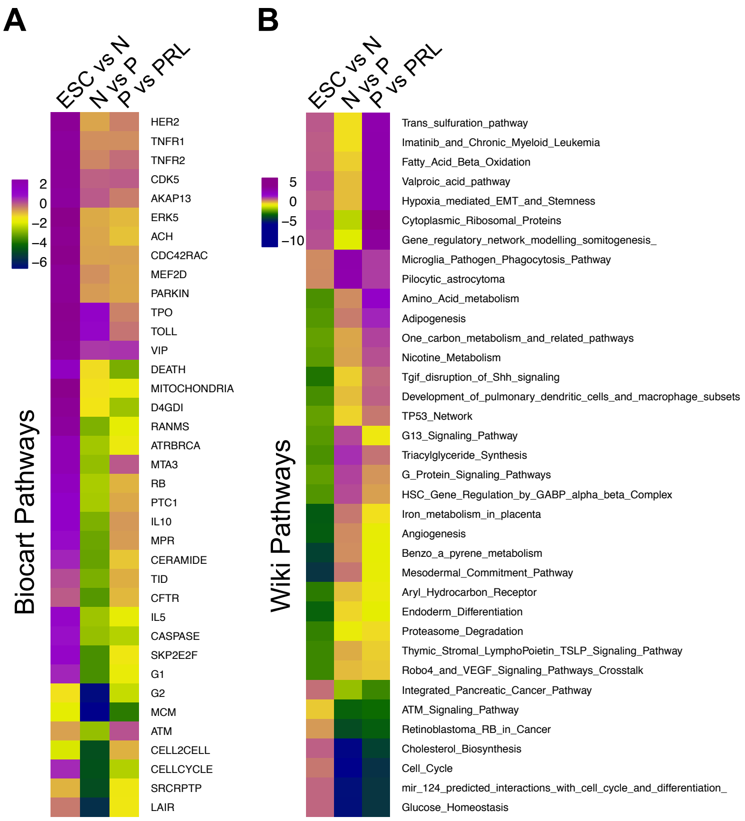
A
ESC vs N
N vs P
P vs PRL
Biocart Pathways
2
0
−2
−4
−6
HER2
TNFR1
TNFR2
CDK5
AKAP13
ERK5
ACH
CDC42RAC
MEF2D
PARKIN
TPO
TOLL
VIP
DEATH
MITOCHONDRIA
D4GDI
RANMS
ATRBRCA
MTA3
RB
PTC1
IL10
MPR
CERAMIDE
TID
CFTR
IL5
CASPASE
SKP2E2F
G1
G2
MCM
ATM
CELL2CELL
CELLCYCLE
SRCRPTP
LAIR
B
ESC vs N
N vs P
P vs PRL
Wiki Pathways
5
0
−5
−10
Trans_sulfuration_pathway
Imatinib_and_Chronic_Myeloid_Leukemia
Fatty_Acid_Beta_Oxidation
Valproic_acid_pathway
Hypoxia_mediated_EMT_and_Stemness
Cytoplasmic_Ribosomal_Proteins
Gene_regulatory_network_modelling_somitogenesis_
Microglia_Pathogen_Phagocytosis_Pathway
Pilocytic_astrocytoma
Amino_Acid_metabolism
Adipogenesis
One_carbon_metabolism_and_related_pathways
Nicotine_Metabolism
Tgif_disruption_of_Shh_signaling
Development_of_pulmonary_dendritic_cells_and_macrophage_subsets
TP53_Network
G13_Signaling_Pathway
Triacylglyceride_Synthesis
G_Protein_Signaling_Pathways
HSC_Gene_Regulation_by_GABP_alpha_beta_Complex
Iron_metabolism_in_placenta
Angiogenesis
Benzo_a_pyrene_metabolism
Mesodermal_Commitment_Pathway
Aryl_Hydrocarbon_Receptor
Endoderm_Differentiation
Proteasome_Degradation
Thymic_Stromal_LymphoPoietin_TSLP_Signaling_Pathway
Robo4_and_VEGF_Signaling_Pathways_Crosstalk
Integrated_Pancreatic_Cancer_Pathway
ATM_Signaling_Pathway
Retinoblastoma_RB_in_Cancer
Cholesterol_Biosynthesis
Cell_Cycle
mir_124_predicted_interactions_with_cell_cycle_and_differentiation_
Glucose_Homeostasis

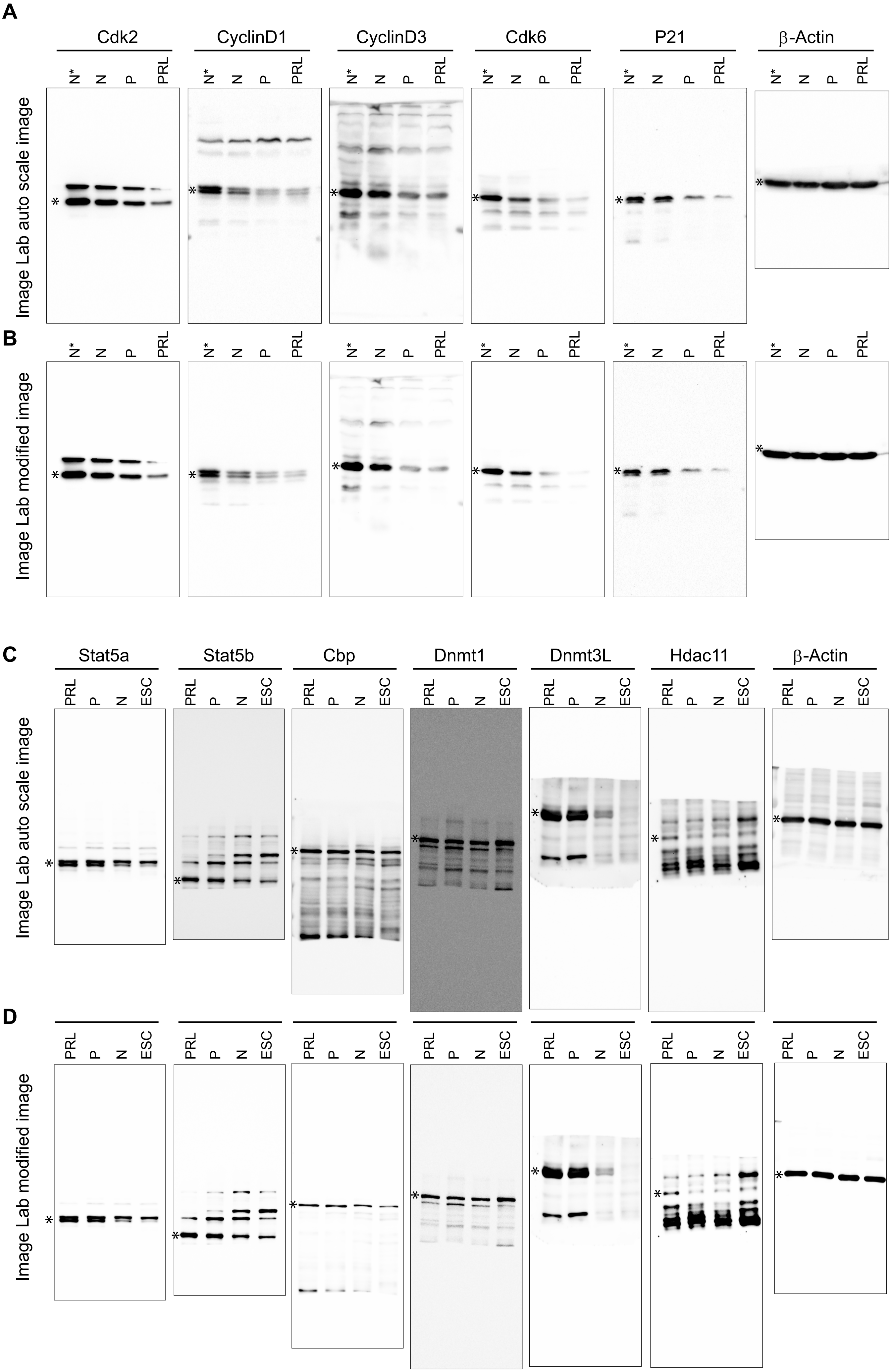
A
Cdk2
CyclinD1
CyclinD3
Cdk6
P21
β-Actin
N* N P PRL
Image Lab auto scale image
B
N* N P PRL
Image Lab modified image
C
Stat5a
Stat5b
Cbp
Dnmt1
Dnmt3L
Hdac11
β-Actin
PRL P N ESC
Image Lab auto scale image
D
PRL P N ESC
Image Lab modified image

Supplementary Figure Legends:

Supplementary Fig.S1. Comparative pathway analysis of differentially expressed genes between ESC and HC11 cell-types. Heatmap showing enrichment score (Z-score) of up or downregulated genes using **(A)** BioCarta and **(B)** Wiki pathway tools between ESC vs. N, N vs. P and P vs. PRL treated HC11 cells. Z-score >2 denote significantly upregulated and <2 denotes significantly downregulated.

Supplementary Fig.S2. Full-length immunoblotting images of respective protein markers **(A)** and contrast adjusted images **(B)** of cell cycle regulators. Similarly, differentially expressed TFs and ERs in auto scale **(C)** and contrast adjusted images **(D).** The molecular weight (kDa) of a marker is highlighted in asterisk (*).
